# 脉冲直流电喷雾质谱法快速检测毛发中4种苯丙胺类药物

**DOI:** 10.3724/SP.J.1123.2023.04002

**Published:** 2023-12-08

**Authors:** Kun MI, Wentian ZHANG, Luhong WEN, Jin WANG

**Affiliations:** 1.国际竹藤中心,北京100102; 1. International Centre for Bamboo and Rattan, Beijing 100102, China; 2.宁波华仪宁创智能科技有限公司,浙江宁波315200; 2. China Innovation Instrument Co., Ltd., Ningbo 315000, China; 3.宁波大学高等技术研究院,浙江宁波315211; 3. Research Institute of Advanced Technologies, Ningbo University, Ningbo 315211, China

**Keywords:** 脉冲直流电喷雾电离, 苯丙胺类药物, 毛发, 快速检测, pulsed direct current electrospray ionization (Pulsed-DC-ESI), amphetamine-type drugs, hair, rapid detection

## Abstract

苯丙胺类药物具有成瘾性和致幻性的特点,滥用问题严重。毛发具有易保存、稳定、可检测时限长的优势,是追溯长期药物滥用史的重要样本。针对现有苯丙胺类药物快速检测技术的不足,以及实验室仪器无法应用于现场快速检测等问题,本文将脉冲直流电喷雾电离(pulsed direct current electrospray ionization, Pulsed-DC-ESI)与便携式质谱仪联用,建立了毛发中4种苯丙胺类药物(苯丙胺、甲基苯丙胺、3,4-亚甲基二氧基苯丙胺、3,4-亚甲双氧基甲基苯丙胺)的快速检测方法。毛发以甲醇为提取溶剂,用研磨法提取,离心后取上清液,直接经Pulsed-DC-ESI离子化后进行质谱分析。实验优化了离子源参数,包括喷雾毛细管尖端内径、电极与待测溶液间距、喷雾电压等。结果表明,该方法可在20 s内完成4种供试药物的同时检测。苯丙胺的线性范围为1~25 ng/mg,甲基苯丙胺的线性范围为1~100 ng/mg,其他两种药物的线性范围为1~50 ng/mg,线性相关系数均大于0.99, 4种苯丙胺类药物的检出限为0.1~0.2 ng/mg,定量限为1 ng/mg,加标回收率为86.6%~114.7%,日内精密度为4.14%~7.34%,日间精密度为3.71%~8.43%。将该方法应用于检测机构提供的2000份实际样品的快速检测,其中有5例甲基苯丙胺阳性样本被检出,与传统司法鉴定方法的结果一致。该方法具有检出限低、准确和快速等特点,适合于毛发中4种苯丙胺类药物的快速检测,同时解决了大型质谱仪无法便携移动和传统快检方法定量能力不足的问题,有助于提升一线缉毒现场对苯丙胺类药物的快速检测能力。

## Abstract (en)

Amphetamine-type drugs are synthetic compounds with an amphetamine parent structure. These compounds cause addiction, central nervous system excitation, and hallucinations. The number of drug users worldwide has gradually increased because amphetamine-type drugs can be synthesized in a simple and artificial manner. The current methods for anti-drug screening and toxicant identification are limited by the large quantity and variety of the drug analytes and long detection times. Thus, the development of broad-spectrum, rapid, and high-throughput detection methods is an urgent necessity. In addition, conventional amphetamine-type drug test samples, such as blood and urine, are only suitable for short-term drug identification. Hair has the advantages of easy preservation, stability, and a long detection window, which can compensate for the deficiencies of body-fluid-based test materials. Hair samples can reflect long-term drug use, which is beneficial for tracing drug sources, and has become an important means of providing evidence in court. Because most laboratory instruments are unable to perform the rapid on-site detection of amphetamine-type drugs in hair, establishing a high-throughput, qualitative and quantitative rapid on-site detection method is necessary. In this study, pulsed direct current electrospray ionization (Pulsed-DC-ESI) coupled with mass spectrometry was used for the rapid detection of four amphetamine-type drugs (amphetamine, methamphetamine, 3,4-methylenedioxyamphetamine, and 3,4-methylenedioxymethamphetamine) in hair. Methanol was used as the extraction solvent, and the grinding method was used for extraction. The pretreatment process included cutting, grinding, and centrifugation. The pretreatment time for each sample was about 10 min. Multiple samples could be processed in batches, greatly improving the efficiency of analysis. Pulsed-DC-ESI is an ambient ionization technology that can be conducted via direct injection without chromatographic separation. The tip of the spray capillary tube was immersed 1 cm below the surface of the sample solution to allow absorption via the capillary effect. When the spray capillary tube contained 1 μL of the sample solution, detection was performed. Pulsed-DC-ESI generates an electrospray at the same frequency as the mass spectrum, thereby avoiding the problem of sample wastage, which often occurs in traditional ESI. The portable mass spectrometer used for analysis is a linear ion trap mass spectrometer. The parameters of Pulsed-DC-ESI, such as the inner diameter of spray capillary tip, spray voltage, and distance between electrode and solution, were optimized based on the mass spectral responses of the amphetamine-type drugs. The optimized ion source conditions included a inner diameter of spray capillary tip of 25 μm, spray voltage of 2 kV, and the distance between electrode and solution of 20 mm. The optimal sample solvent was methanol. The optimized method can achieve simultaneous detection of the four amphetamine-type drugs within 20 s. The linear ranges of amphetamine, methamphetamine, and the two other drugs were 1-25, 1-100, and 1-50 ng/mg, respectively. The limits of detection and quantification of the four drugs in hair were 0.1-0.2 and 1 ng/mg, respectively. All linear correlation coefficients were greater than 0.99, and the average spiked recoveries were 86.6%-114.7%. The intra-day precisions were 4.14%-7.34%, and the inter-day precisions were 3.71%-8.43%. The proposed method was used to screen 2000 samples provided by various testing institutions. A total of five samples were positive for methamphetamine, which is consistent with the results of conventional forensic identification methods. Thus, the developed method can be used for the rapid detection of amphetamine-type drugs.

苯丙胺类药物是以苯丙胺(AM)为母体结构的一类化合物,具有成瘾性和致幻性。目前,苯丙胺类药物滥用问题严重,而现有的快速检测手段存在不足,如检测的广谱性、快捷性和便携性等。常规苯丙胺类药物的检测材料(如血液、尿液)仅适用于短期的人体内药物鉴定。毛发是追溯长期药物滥用史的重要样本,具有易保存、稳定、可检测时限长的优势,弥补了体液检测材料的不足^[1]^,是重要的法庭证据。

目前,国内外有关苯丙胺类药物的检测方法包括气相色谱-质谱联用法、液相色谱-质谱联用法、免疫分析法等。色谱-质谱联用法是毒品检测的金标准,韦棋等^[2]^用气相色谱-质谱联用法测定毛发中的6种苯丙胺类药物,线性范围为0.5~5.0 ng/mg。苏佳丽等^[3]^用超高效液相色谱-质谱联用法测定毛发中的常见毒品,其中苯丙胺类药物的线性范围为0.1~40 ng/mg。据广西公安厅物证鉴定中心统计,苯丙胺类药物滥用人员的阳性尿液中,苯丙胺类药物的平均含量为29~13530 ng/mL^[4]^;据我国公安部统计,苯丙胺类药物滥用人员的毛发中,苯丙胺类药物的平均含量为1.98~15.09 ng/mg^[5]^。传统的苯丙胺类药物快速检测技术主要以尿液为样本,采用胶体金免疫检测法^[6]^。Cheong等^[7]^使用荧光偏振免疫分析法测定头发中的甲基苯丙胺(MA),对加标样品(甲基苯丙胺含量0.5 ng/mg)的阳性检出率可达100%,其检测灵敏度与色谱仪器相当,但该方法的样品前处理涉及长达4 h的酶消化过程。

敞开式质谱技术的出现促进了质谱方法在现场快速检测中的应用。敞开式质谱技术的特点是在开放大气压环境下对待测物进行解吸和电离,待测物离子化后进入质谱仪分析;与传统的色谱-质谱技术相比,敞开式质谱技术可以省略色谱分离环节,且无需复杂的样品前处理^[8]^。敞开式质谱的代表性技术包括解析电喷雾电离(desorption electrospray ionization, DESI)^[9]^、实时直接分析(direct analysis in real time, DART)^[10]^、纸喷雾电离(paper spray ionization, PSI)^[11]^等,敞开式质谱技术已广泛应用于公共安全^[12]^、食品安全^[13]^等领域。Teunissen等^[14]^开发了基于纸喷雾电离的全血中苯丙胺快速定量方法,检出限为15~50 ng/mL。熊士领等^[15]^利用探针直接电离实现了尿液中5种毒品的快速检测,其中甲基苯丙胺的检出限为0.5 ng/mL,可满足尿液司法鉴定检测标准要求(1 ng/mL)^[16]^。脉冲直流电喷雾电离(pulsed direct current electrospray ionization, Pulsed-DC-ESI)^[17]^作为一种新型敞开式直接电离技术,其电喷雾是通过在靠近待测溶液但不与待测溶液接触的电极上施加高压直流电产生的。与传统质谱相比,Pulsed-DC-ESI质谱无需复杂的样品前处理,样品消耗少,无需载气辅助,离子可直接进入质谱仪,大大提高了检测效率^[18]^。

本研究将脉冲直流电喷雾与便携式质谱仪联用,通过优化Pulsed-DC-ESI参数,明确了影响Pulsed-DC-ESI稳定性和离子化效率的关键因素,建立了一种人体毛发样品中4种苯丙胺类药物的快速检测方法,并进行了方法学验证,旨在为司法部门现场快速检测苯丙胺类药物提供技术支撑。

## 1 实验部分

### 1.1 仪器、试剂与材料

CRAIV-110便携式质谱仪、PDESI-100脉冲直流电喷雾离子源(宁波华仪宁创智能科技有限公司); B100-70-10微电极玻璃毛细管(美国Sutter公司); PC-100程控垂直拉制仪(日本Narishige公司); Vortes-5涡旋仪(海门市其林贝尔仪器制造有限公司); Hico台式高速微量离心机(生工生物工程(上海)股份有限公司); JXFSTPRP-CLN研磨仪(上海净信实业发展有限公司); SECURA125-1CN电子天平(德国赛多利斯公司)。

甲醇、乙腈、乙酸乙酯(HPLC级,美国TEDIA公司);AM、MA、3,4-亚甲基二氧基苯丙胺(MDA)和3,4-亚甲双氧甲基苯丙胺(MDMA)标准品(上海刑事科学技术研究院,质量浓度1 mg/mL)。实验所用空白毛发是经过Agilent 1260 Infinity Ⅱ液相色谱-Agilent 6470B三重四极杆质谱联用仪检验后无毒的多地、多人毛发混合样本,由宁创检验(宁波)有限公司提供。

### 1.2 标准溶液的配制

分别精确移取125 μL的4种标准品至1 mL样品瓶中,以甲醇为溶剂配制成质量浓度为125 μg/mL的混合标准储备液。取适量混合标准储备液,用甲醇稀释成系列质量浓度(0.5、1、2.5、5、25、50、100 μg/mL)的混合标准溶液。

### 1.3 样品前处理

称取25 mg毛发样品(称量精度0.1 mg),剪至长度小于1 cm的片段,放入研磨管中(含不锈钢研磨珠),加入300 μL甲醇,在65 Hz条件下研磨6 min,然后将提取液在13000 r/min下离心3 min;之后把毛细管尖端浸至提取液液面以下1 cm,利用毛细效应吸取提取液(约1 μL),进质谱检测。

### 1.4 加标样品的制备

按照1.3节方法对毛发样品进行前处理后,加入适量的混合标准溶液,制备成不同含量(0.1、0.2、1、5、10、25、50、100、200 ng/mg)的加标样品,备用。

### 1.5 仪器条件

质谱条件:锥口温度200 ℃,离子通道内径0.37 mm,采样锥孔长度100 mm,喷雾电压2 kV,正离子结合多反应监测(MRM)模式采集数据。4种苯丙胺类化合物的质谱采集参数见表1。

**表1:** 4种苯丙胺类化合物的质谱参数

Compound	Parent ion (*m/z*)	Product ions (*m/z*)	Fragmentation voltage/V
Amphetamine (AM)	136	119^*^, 91	2.0
Methamphetamine (MA)	150	119, 91^*^	1.5
3,4-Methylene dioxy amphetamine (MDA)	180	163^*^, 135	2.0
3,4-Methylene dioxy methamphetamine (MDMA)	194	163^*^, 105	2.0

* Quantitative ion.

脉冲直流电喷雾离子源:喷雾毛细管为硼硅酸盐玻璃毛细管,外径1 mm,内径0.75 mm,长度55 mm;喷雾毛细管尖端内径25 μm,尖端玻璃壁厚约12 μm。脉冲直流电喷雾离子源的装置示意图见图1。

**图1:**
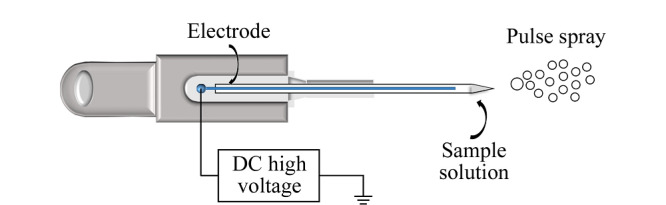
脉冲直流电喷雾离子源的装置示意图

## 2 结果与讨论

### 2.1 离子源条件优化

离子源条件是质谱信号稳定、检测灵敏度高的重要保障。为使Pulsed-DC-ESI-便携式质谱仪达到最佳检测状态,对脉冲直流电喷雾离子源的各组成部分进行单因素实验。以4种苯丙胺类化合物的离子强度作为评价指标,考察喷雾毛细管尖端内径(25、50 μm)、电极与待测溶液间距(*d*, 0~30 mm)以及喷雾电压(1~5 kV)等因素对质谱稳定性和离子化效率的影响,每个实验重复6次,每次更换不同的喷雾毛细管。

#### 2.1.1 喷雾毛细管尖端内径优化

检测灵敏度与毛细管尖端内径有关,一般认为,毛细管尖端内径越小,形成泰勒锥中单位体积的电荷密度越大,越有利于库仑爆炸的形成。根据预实验结果,比较了不同喷雾毛细管尖端内径(25、50 μm)对离子化效率的影响。在喷雾电压为2 kV、电极与待测溶液间距为20 mm的条件下,对4种苯丙胺类药物混合标准溶液的质谱响应进行测试,结果见图2。由图2可知,当喷雾毛细管尖端内径为25 μm时,4种苯丙胺类化合物均具有较好的质谱响应性和稳定性;当喷雾毛细管尖端内径为50 μm时,质谱响应的稳定性较差。在预实验中,当喷雾毛细管尖端内径为10 μm时,因浸取的样品量较少,会导致无法产生稳定的质谱信号。因此,选择喷雾毛细管尖端内径为25 μm,有助于稳定质谱响应信号。

**图2:**
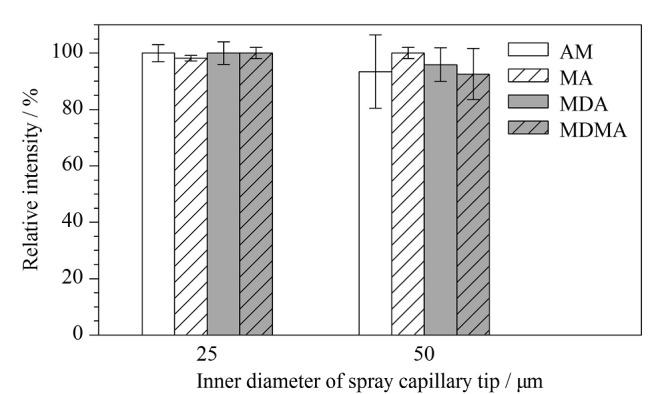
喷雾毛细管尖端内径对4种苯丙胺类化合物质谱响应的影响(*n*=6)

#### 2.1.2 电极与待测溶液的间距优化

Pulsed-DC-ESI的原理是电极尖端产生的感应电场诱导待测溶液极化^[19]^,在正离子模式下,正离子向毛细管尖端迁移,积累到一定程度会产生电喷雾;同时,待测溶液中也会积累负离子,需通过氧化还原反应实现溶液的电中性,产生连续的电喷雾^[20]^。电极与待测溶液的间距决定了电极尖端产生的电场在待测溶液上的强度,实验考察了电极与待测溶液间距对4种苯丙胺类药物质谱响应的影响。在喷雾电压为2 kV、喷雾毛细管尖端内径为25 μm的条件下,对4种苯丙胺类药物混合标准溶液进行测试,结果如图3所示。

**图3:**
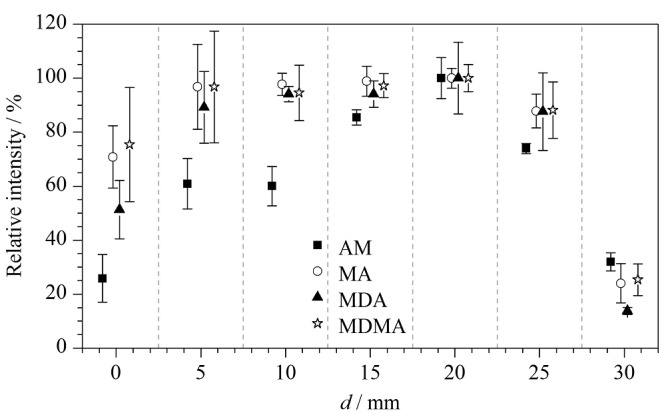
电极与待测溶液间距对4种苯丙胺类化合物质谱响应的影响(*n*=6)

由图3可知,当电极与待测溶液间距为20 mm时,4种苯丙胺类药物表现出较好的质谱响应。预实验发现,当电极直接接触待测溶液时,会出现溶液瞬间大量喷出的现象,导致软件无法采集完整的质谱信号,且待测溶液附着在电极表面,会对电极造成污染,需清洗电极才能重复使用。因此,本研究将电极与待测溶液的间距设置为20 mm。

#### 2.1.3 喷雾电压优化

适当的喷雾电压有利于质谱信号的产生。在喷雾毛细管尖端内径为25 μm、电极与待测溶液间距为20 mm的条件下,比较了不同喷雾电压(1~5 kV)对4种苯丙胺类化合物信号强度的影响,结果见图4。由图4可知,当喷雾电压为2 kV时,可产生强度较高且稳定的质谱信号,随着电压增加,AM的质谱信号下降,电压对电离过程并不表现为持续的促进作用。相同溶剂条件下,毛细管喷出带电雾滴的大小与喷雾毛细管尖端的电场强度呈正相关^[20]^,电场强度越大,喷雾液滴越大。当待测溶液体积不变时,带电雾滴越大,电喷雾的持续时间越短。因此,用于计算离子有效强度对应的时长不足,质谱软件无法采集足够多的质谱信号。实验过程发现,当较高的电压施加在电极上时,质谱响应信号存在不稳定的现象。因此,确定喷雾电压为2 kV。

**图4:**
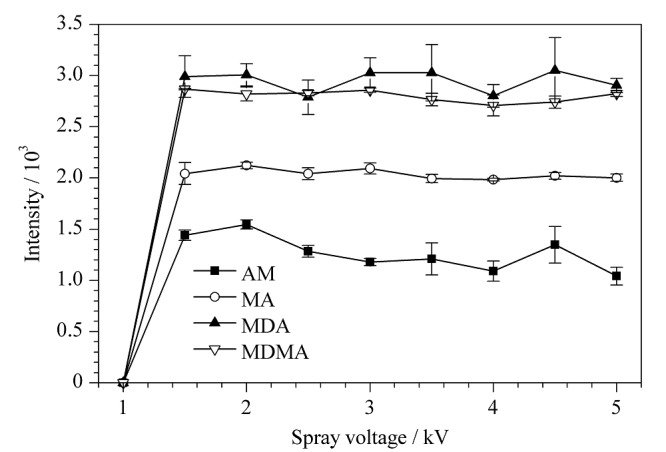
喷雾电压对4种苯丙胺类化合物质谱响应的影响(*n*=6)

### 2.2 样品溶剂优化

不同溶剂具有不同的理化性质,如黏度、表面张力等,这些性质会对样品的离子化产生影响。本研究分别以3种不同溶剂(甲醇、乙腈和乙酸乙酯)溶解4种苯丙胺类化合物,比较了不同溶剂对苯丙胺类药物质谱响应的影响。在喷雾电压为2 kV、喷雾毛细管尖端内径为25 μm、电极与待测溶液间距为20 mm的条件下进行测试。结果如图5所示,4种苯丙胺类化合物在甲醇中均具有较好的质谱信号强度,但在乙腈和乙酸乙酯中,4种苯丙胺类化合物的质谱响应下降,这可能是因为乙腈的质子传输能力比甲醇低,导致苯丙胺类化合物的质谱响应较低;当乙酸乙酯作为溶剂时会产生质谱响应信号延迟和电喷雾不稳定的现象,这可能是因为乙酸乙酯的极性较弱^[21]^,需要较长时间的电场诱导才能极化,不利于电喷雾的产生。因此,本实验最终选择甲醇为样品的溶解溶剂。在优化的条件下,4种苯丙胺类化合物的质谱采集参数见表1,二级质谱图见图6。

**图5:**
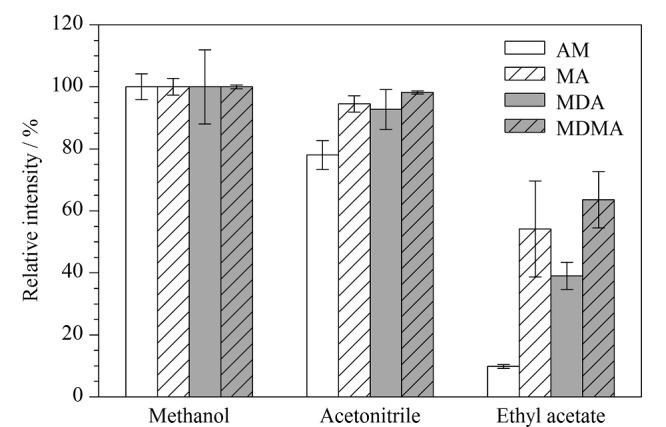
不同溶解溶剂对4种苯丙胺类化合物质谱响应的影响(*n*=6)

**图6:**
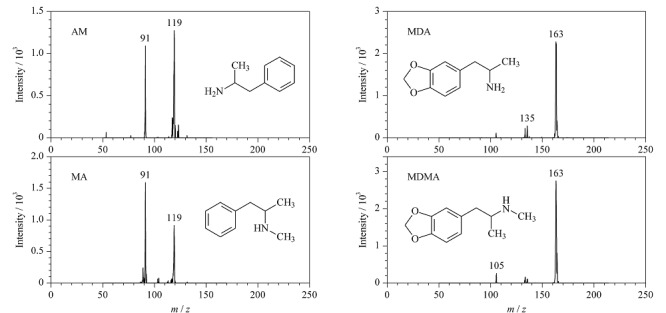
4种苯丙胺类化合物(0.1 μg/mL)的二级质谱图

### 2.3 方法学考察

#### 2.3.1 基质效应考察

对空白毛发提取液直接添加苯丙胺类药物混合标准溶液,测定其基质效应(ME)^[22]^。基质效应=空白毛发基质提取液加标离子响应强度/对应浓度混合标准溶液离子响应强度×100%。ME大于100%,表示存在基质增强效应;ME小于100%,表示存在基质抑制效应。本方法中4种苯丙胺类药物的基质效应为93.2%~109.8%,结果表明,毛发基质对于目标化合物的检测未产生明显的基质增强或基质抑制效应。

#### 2.3.2 线性范围、检出限和定量限

在优化的条件下,对制备的加标样品(0.1、0.2、1、5、10、25、50、100、200 ng/mg)进样测试。以4种苯丙胺类化合物的含量为横坐标(*x*)、定量离子质谱信号强度为纵坐标(*y*),绘制标准曲线并计算线性回归方程。4种苯丙胺类化合物的线性回归方程和相关系数(*R*)等数据见表2。苯丙胺的线性范围为1~25 ng/mg,甲基苯丙胺的线性范围为1~100 ng/mg,其余两种药物的线性范围为1~50 ng/mg,线性相关系数均大于0.99。以3倍信噪比计算检出限(LOD)、10倍信噪比计算定量限(LOQ)。4种苯丙胺类化合物在毛发基质中的检出限为0.1~0.2 ng/mg,定量限为1 ng/mg,均满足司法鉴定规范^[23]^的检出限阈值要求(0.2 ng/mg)。

**表2:** 4种苯丙胺类化合物的线性回归方程、相关系数、检出限和定量限

Compound	Linear range/(ng/mg)	Linear regression equation	*R*	LOD/(ng/mg)	LOQ/(ng/mg)
AM	1-25	*y*=11.770*x*+0.7589	0.9973	0.1	1
MA	1-100	*y*=19.082*x*-1.8028	0.9952	0.1	1
MDA	1-50	*y*=36.823*x*-3.0460	0.9985	0.2	1
MDMA	1-50	*y*=38.443*x*+6.8327	0.9987	0.2	1

*y*: MS signal intensity; *x*: content, ng/mg.

#### 2.3.3 方法精密度

以4种苯丙胺类化合物的空白基质加标溶液(50 ng/mg)为样品、质谱信号强度的相对标准偏差(RSD)为指标,考察方法的日内和日间精密度。供试样品溶液在一日内连续重复进样6次,测得日内精密度;连续3天,每天重复进样6次,测得日间精密度,结果见表3。如表3所示,4种苯丙胺类药物的日内精密度为4.14%~7.34%(*n*=6),日间精密度为3.71%~8.43%(*n*=6)。4种苯丙胺类化合物的日内精密度和日间精密度均在10%以内,表明该方法具有良好的稳定性。

**表3:** 4种苯丙胺类化合物的日内和日间精密度(*n*=6)

Compound	Intra-day RSD/%	Inter-day RSD/%
AM	4.14	8.34
MA	5.96	3.71
MDA	7.34	8.43
MDMA	6.25	7.61

#### 2.3.4 方法准确度

以加标回收率为指标考察实验的准确度,准确称量空白毛发样品3份,分别加入低、中、高3个水平的苯丙胺混合标准溶液,测定4种苯丙胺类药物的含量,计算加标回收率及其相对标准偏差,结果见表4。如表4所示,4种苯丙胺类化合物在毛发中的加标回收率为86.6%~114.7%,相对标准偏差为1.4%~10.8%,说明该方法的准确度能够满足苯丙胺类化合物的检测要求。空白毛发加标(10 ng/mg)样品的质谱图见图7。

**表4:** 4种苯丙胺类化合物在空白毛发基质中的加标回收率及相对标准偏差(*n*=6)

Compound	Spiked level/ (ng/mg)	Found/ (ng/mg)	Recovery/ %	RSD/ %	Compound	Spiked level/ (ng/mg)	Found/ (ng/mg)	Recovery/ %	RSD/ %	
AM	1	1.0	96.0	10.8	MDA	1	0.9	86.6	9.8	
	10	9.8	97.9	9.6		25	21.8	87.1	1.4	
	25	25.9	103.4	5.1		50	56.1	112.1	7.5	
MA	1	1.2	114.7	4.0	MDMA	1	0.9	87.1	8.9	
	50	46.6	93.3	7.3		25	23.0	92.0	4.8	
	100	103.6	103.6	6.3		50	52.0	103.9	6.8	

**图7:**
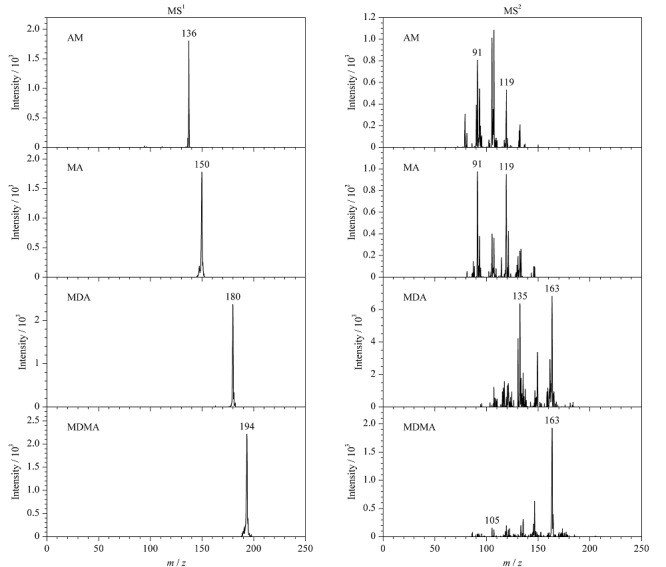
4种苯丙胺类化合物加标(10 ng/mg)毛发样品的一级和二级质谱图

### 2.4 与其他方法的比较

司法鉴定规范(SF/Z JD0107025-2018)^[23]^的前处理方法如下:将毛发样品依次用适量的水和丙酮振荡洗涤两次,晾干后剪成约1 mm的片段,置于冷冻研磨仪中粉碎,至呈粉末状。称取毛发粉末20 mg,加入1.0 mL甲醇,冰浴超声30 min,离心后移取上清液,于60 ℃水浴空气流下吹干,再用100 mL甲醇复溶,进仪器分析。与司法鉴定规范的样品前处理相比,本方法省略了毛发样品的干燥、超声和浓缩等步骤,提高了检测效率。司法鉴定规范的单一样本处理时长约为1 h,本方法对单一样品处理时间约为10 min,多样品可批量处理操作,效率更高,检测单一样品用时小于20 s。传统免疫胶体金法的灵敏度较低,甚至无法达到毛发中毒品检测阈值的要求。因此,本方法具有操作简便、快捷和准确的特点,能够满足4种苯丙胺类化合物的司法鉴定要求,有助于促进我国毒品快检能力建设。

### 2.5 实际样品检测

用Pulsed-DC-ESI-便携式质谱仪对来自浙江省某地公安局的实际毛发样品进行检测,以验证本方法的适用性。在2000份待测样本中,检出甲基苯丙胺阳性样本5例,其余均为阴性样本,本方法检出的阳性样本数与司法鉴定方法的检测结果一致。此外,本方法未在甲基苯丙胺阳性样本中检测出甲基苯丙胺的代谢产物苯丙胺,这可能与甲基苯丙胺在毛发中主要以原药形态存在有关,且苯丙胺在毛发中的含量较少,有研究^[5]^报道阳性样本中苯丙胺与甲基苯丙胺含量的比率为0.015~0.384。

## 3 结论

本研究基于Pulsed-DC-ESI-便携式质谱仪联用平台,优化了Pulsed-DC-ESI离子源中电极与待测溶液的间距、喷雾电压、样品溶剂以及喷雾毛细管尖端内径等参数,开发了人体毛发中4种苯丙胺类药物的快速检测方法。经方法学验证,该方法具有检出限低、准确和快速等特点。在实际样品检测中,本方法无需复杂的前处理和色谱分离过程,适合于毛发中4种苯丙胺类药物的快速检测分析。针对大型质谱仪无法便携移动、样品前处理耗时长、传统快检方法定量能力不足等问题,本方法提供了有效的替代方案,能够同时满足快速检测和定性、定量的需求,有助于提高一线缉毒现场对毒品的快速检测能力。
